# Early‐life telomere length predicts life‐history strategy and reproductive senescence in a threatened wild songbird

**DOI:** 10.1111/mec.16981

**Published:** 2023-05-12

**Authors:** Fay Morland, John G. Ewen, Mirre J. P. Simons, Patricia Brekke, Nicola Hemmings

**Affiliations:** ^1^ Department of Biosciences University of Sheffield Sheffield UK; ^2^ Institute of Zoology, Zoological Society of London London UK; ^3^ Department of Anatomy University of Otago Dunedin New Zealand

**Keywords:** fitness, life history, reproduction, senescence, telomeres

## Abstract

Telomeres are well known for their associations with lifespan and ageing across diverse taxa. Early‐life telomere length can be influenced by developmental conditions and has been shown positively affect lifetime reproductive success in a limited number of studies. Whether these effects are caused by a change in lifespan, reproductive rate or perhaps most importantly reproductive senescence is unclear. Using long‐term data on female breeding success from a threatened songbird (the hihi, *Notiomystis cincta*), we show that the early‐life telomere length of individuals predicts the presence and rate of future senescence of key reproductive traits: clutch size and hatching success. In contrast, senescence of fledging success is not associated with early‐life telomere length, which may be due to the added influence of biparental care at this stage. Early‐life telomere length does not predict lifespan or lifetime reproductive success in this species. Females may therefore change their reproductive allocation strategy depending on their early developmental conditions, which we hypothesise are reflected in their early‐life telomere length. Our results offer new insights on the role that telomeres play in reproductive senescence and individual fitness and suggest telomere length can be used as a predictor for future life history in threatened species.

## 1 INTRODUCTION

Telomeres have been likened to a ‘biological clock’, with the gradual shortening of telomeres at each cell division ticking down to senescence and eventual cell death. This analogy has been consolidated by the many studies that have investigated the relationship between telomere length and ageing in humans (Cawthon et al., 2003), domestic animals, (Seeker et al., 2018; Sohn & Subramani, 2014), model organisms (Haussmann & Vleck, 2002; Heidinger et al., 2012; Muñoz‐Lorente et al., 2019) and wild populations across taxa (Cherdsukjai et al., 2020; Froy et al., 2021; Sánchez‐Montes et al., 2020) including birds (Bichet et al., 2020; Tricola et al., 2018; Vedder et al., 2021); concluding that telomere length shortens with increasing age and predicts aspects of ageing, including lifespan. However, the rate of telomere shortening varies between and within individuals; for example, with age, body mass and growth rates (Barrett et al., 2013; Hall et al., 2004), this lack of constancy in telomere shortening calls into question the appropriateness of the ‘biological clock’ metaphor. In addition, it is unclear whether telomere shortening actually causes senescence, or whether the association between these two processes is simply indirect (Horn, 2008; Simons, 2015). Comparative analysis across species is contradictory, with a negative relationship between telomere length and maximum lifespan in mammals (Pepke & Eisenberg, 2021), and the opposite relationship across bird species, where telomere shortening is a strong predictor of a species' lifespan (Tricola et al., 2018). Despite considerable research effort in this area, it is evident that the role of telomeres in ageing remains elusive.

Telomere length has not only been associated with lifespan but also with the second pillar of fitness: reproduction. Current theory on the evolution of ageing, including the disposable soma theory (Kirkwood & Holliday, 1979; Kirkwood et al., 1991) and the principle of allocation (Cody, 1966), predicts that reproduction should trade‐off with longevity and future survival. There is empirical evidence to support these theories; for example, a cross‐taxa meta‐analysis (Lemaître et al., 2015) and both experimental and observational studies on birds, which find that higher reproductive output leads to increased mortality rate (Boonekamp, Salomons, et al., 2014) and reduced future clutch size (Gustafsson & Pärt, 1990). If reproductive effort accelerates senescence, telomere shortening may provide a mechanism; increased reproductive effort has been associated with shorter telomeres in multiple species (e.g. common tern, *Sterna hirundo* (Bauch et al., 2013), blue tit, *Cyanistes caeruleus* (Sudyka et al., 2014, 2019), mice, *Mus musculus* (Kotrschal et al., 2007), reviewed in Sudyka, 2019). However, an experimental study on zebra finches, *Taeniopygia guttata*, which manipulated early‐life reproductive effort, found that while engaging in reproduction led to a significant reduction in telomere length (compared with nonbreeding individuals), this difference was only apparent during the focal breeding attempt, with an absence of long‐term effects of breeding on telomere length (Heidinger et al., 2012). There is also recent meta‐analytic evidence questioning the classic theories of ageing, arguing that variation in individual quality masks the fitness costs of the reproduction–survival trade‐off (Winder et al., 2022). Questions therefore remain about the long‐term or lifetime effects of reproduction on telomeres and ageing.

Developmental conditions can shape future life‐history decisions (Auer, 2010), individual quality (Hamel et al., 2009) and rate of reproductive senescence (Balbontín & Møller, 2015; Nussey et al., 2007), as predicted by the ‘silver spoon’ hypothesis (Grafen, 1988), and these long‐term effects have also been linked to telomere length (Eastwood et al., 2019; Heidinger et al., 2012). Early‐life conditions impact telomere length; for example, early‐life telomere length is related to the age and condition of parents (Asghar et al., 2015; Bennett et al., 2021; Marasco et al., 2019) and is negatively impacted by environmental effects during development, including stress (Boonekamp, Mulder, et al., 2014; Herborn et al., 2014; Stier et al., 2020), adverse weather (Eastwood et al., 2022) and heightened sibling competition (Cram et al., 2017; Nettle et al., 2013). Early‐life telomere length is therefore a likely biomarker of an individual's longer‐term ‘quality’, shaped by both its inherited characteristics and its developmental conditions. Indeed, longer early‐life telomere length predicts a longer lifespan (Heidinger et al., 2012; van Lieshout et al., 2019), lower mortality risk across life (Eastwood et al., 2023) and, as a consequence, higher lifetime reproductive success (Eastwood et al., 2019; Heidinger et al., 2021). Few studies have examined these lifelong implications of early‐life telomere length, and the life‐history consequences of early‐life telomere length for an individual, in particular, are not well understood. Specifically, to our knowledge, there have been no studies on the effect of early‐life telomere length on the rate of senescence of reproductive traits.

Reproductive senescence is widespread in mammals and birds (Lemaître & Gaillard, 2017; Vágási et al., 2021). An individual's rate of actuarial senescence has been shown to co‐vary with reproductive investment (Nussey et al., 2006; Reed et al., 2008) and environmental conditions during early life (Balbontín & Møller, 2015). Different reproductive traits can also vary in how they senesce (Berger et al., 2015; Hayward et al., 2013). Fitness will thus depend on how individual traits contribute to fitness and how these traits senesce. Hatching success, clutch size, fledgling production, breeding duration, laying date and number of breeding attempts are all traits that can significantly contribute to fitness, but can vary in their relative importance (Merilä & Sheldon, 2000; Teplitsky et al., 2009). We currently have limited understanding of the mechanisms that link life‐history traits to ageing and fitness. Telomeres provide an attractive potential window into these mechanisms, but studies on telomeres covering the entire reproductive lifespan of individuals are lacking (Sudyka, 2019) and few studies take multiple reproductive traits into account when studying reproductive senescence (Vágási et al., 2021).

Here, we examine how early‐life telomere length relates to reproductive senescence and lifetime reproductive success in a threatened species of passerine bird, the hihi (*Notiomystis cincta*). The early‐life telomere lengths of 75 females were measured using qPCR (Cawthon, 2002), with blood samples taken at 21 days old. We test the relationship between early‐life telomere length and, (i) patterns of senescence (postpeak decline) in multiple reproductive traits: clutch size, hatching success and fledging success, and (ii) four measures of lifetime reproductive success: the number of eggs laid, eggs hatched, chicks fledged and recruits produced and (iii) lifespan.

## 2 MATERIALS AND METHODS

### 2.1 Study system and data collection

This study was carried out on a managed population of hihi on Tiritiri Matangi Island in northern New Zealand. The population is closed with no recorded migration of birds to mainland New Zealand or other islands, allowing for accurate estimations of natural lifespan. Hihi have a promiscuous breeding system, with no mate fidelity and high levels of extra‐pair paternity (70%; Brekke et al., 2013). Males establish territories prior to the breeding season, which may contain the nests of multiple females (but usually just one), and contribute to the provisioning of the chicks of females within their territory (Ewen & Armstrong, 2000). Hihi will begin breeding in the first season after they fledge, producing an average of 2 successful clutches per breeding season. Each individual bird is colour‐ringed for identification purposes and has a blood sample taken at 21 days old. Hihi use provided nest boxes and during the breeding season (September–February) accurate dates for laying, hatching, and fledging, clutch size, hatching success and fledging success are recorded for all nests. Offspring are assigned a cohort depending on the breeding season within which they hatched. Biannual constant effort population surveys: one prebreeding survey in September and one postbreeding season in February, allows the monitoring of survival, lifespan and nonbreeding individuals (Thorogood et al., 2013). A total of 75 females were included in the analysis, including data on 344 clutches (an average of 4.5 clutches per individual). Females were distributed across nine cohorts spanning the years 2005–2013. For this study, hatching success was measured as the proportion of eggs in a clutch that hatched and fledging success as the proportion of nestlings that fledged. Age of the female at the time of laying was the number of days since the female fledged. The 75 females included in all analyses were recorded as ‘dead’ because the last date they were observed in the breeding or census records was more than 2 years before the onset of the latest breeding season included in the analysis. Lifespan was estimated as the number of days between a female's own fledging date and the last date she was observed in the census or breeding records. For the latter, if a female successfully fledged chicks from her final nest attempt, that fledging date was recorded as the last date the female was observed in the breeding record. Alternatively, if a female did not fledge chicks or hatch eggs during her final nesting attempt, the hatching date or laying date, respectively, of that final nesting attempt was recorded as the last date she was observed in the breeding record.

### 2.2 Telomere analysis

Blood samples were preserved in >80% ethanol and stored at −20°C. DNA was extracted from blood samples using the ammonium acetate method (Bruford et al., 1998), eluted in LowTE and stored frozen at −20°C. Blood was stored for 8–16 years (depending on the cohort of the individual) before DNA extraction and subsequent qPCR was performed on all samples in 2021 To test for variation in DNA quality and degradation across sample ages, gel electrophoresis was performed using a random selection of 40 individual DNA samples representing all 9 cohorts. DNA was of consistently high molecular weight (23 kb or over) and showed no signs of degradation. DNA purity was measured with a NanoDrop, and samples with A260/280 values outside the range of 1.7–2.6 or A260/230 values which fell outside the range 1.7–2.8 underwent a bead clean‐up process using AMPure PB magnetic beads. Samples were subjected to a maximum of 2 bead cleans to achieve the target purity or were otherwise excluded from further analysis. Relative telomere lengths did not differ between those which received a bead clean and those which did not (Figure S1). DNA impurity caused qPCR inefficiency and low repeatability of replicates during trials, possibly because of the use of heparinised capillary tubes for blood sample collection. DNA quantity was assessed and normalized to 1 ng/μL using a Qubit fluorometer.

Telomere analysis was performed using a singleplex quantitative PCR method (Cawthon, 2002; Criscuolo et al., 2009) which provides a relative telomere length or T/S, the ratio of telomere repeat copy number relative to a nonvariable copy number control gene. The qPCR was performed using a QuantStudio 12K Flex Real‐Time PCR System. We used Tel1b (5′‐CGGTTTGTTTGGGTTTGGGTTTGGGTTTGGGTTTGGGTT‐3′) and Tel2b (5′‐GGCTTGCCTTACCCTTACCCTTACCCTTACCCTTACCCT‐3′) to target telomere sequences, and RAG1‐F (5′‐CAGCACATAAACAAAGATCAGGCAG‐3′) and RAG1‐R (5′‐AAGTCCCTGCCTATTGCCCA‐3′) to target the nonvariable copy number control gene; RAG1 is known to be single copy and universal gene in gnathostomes, which includes all tetrapods (Crottini et al., 2012). The RAG1 primer was designed by using BLAST to find identical regions between *Corvus moneduloides* RAG1 mRNA sequence data and hihi RAG1 partial coding region sequence (CDS). *Corvus moneduloides* was selected because it had the highest percentage agreement with the hihi RAG1 sequence (95.96%). The primers were then designed to fall within an identical overlapping region of the *Corvus moneduloides* RAG1 mRNA sequence with the hihi RAG1 partial coding region (CDS) sequence and mRNA sequence data were necessary to allow the designed primer to span an exon–exon junction, ensuring that the primer was specific to cDNA.

Samples were run in triplicate, with telomere and reference primers run on separate plates because the optimum annealing temperature for the 2 primers differed, making a total of eight plates: four containing telomere primers and four containing reference primers and all plates contained a random mix of cohorts. Triplicate samples were run to ensure repeatability of measurements between repeats of the same samples, and the standard deviation in Ct values between triplicates was calculated automatically by the QuantStudio software. Samples were rerun if the standard deviation in Ct values between triplicates exceeded 0.5. The average standard deviation in Ct values between triplicates was 0.14 for RAG1 runs and 0.16 for Tel runs. The average standard deviation for between plates for the serial dilution of the standard sample was 2.5 for RAG1 runs and 1.52 for Tel runs. A between‐plate calibrator was used to account for inter and intraplate variability; every plate included three negative control wells and a 1:4 serial dilution (10, 2.5, 0.625, 0.156 ng) of the same standard (‘golden’) sample in triplicate for each primer. Every reaction contained 1 ng of DNA, 10 μL of SYBR Select Master Mix and 9 μL of a primer/ddH_2_O mix to a concentration of 0.7 μmol for Telomere primers and 0.2 μmol for RAG1 primers. Telomere plates were run using the following method: 50°C (20 s), 95°C (20 s) followed by 40 cycles of 95°C (1 s), 56°C (20 s). Rag1 plates were run with the following method: 50°C (20 s), 95°C (20 s) followed by 40 cycles of 95°C (1 s), 61°C (20 s).

The Ct threshold was set to 1.45 for analysis of Telomere primer reactions and 0.7 for analysis of Rag1 primer reactions. The Ct thresholds were selected as the mean of the automatic thresholds as set by QuantStudio for the multiple plates. Changing the automatic Ct threshold to a standardized Ct threshold did not substantially change the *R* ^2^ or efficiency values of any standard curve (to be within or outside of the acceptable ranges for analysis). Amplification efficiencies, provided by the Applied Biosystems QuantStudio programme which corrects for baseline fluorescence, were 95.5%–96.8% for RAG plates and 102.6%–106% for TEL plates. *R* ^2^ values, also provided in the Applied Biosystems QuantStudio, were 0.99 for all plates. The ‘control average’ was calculated from the standard curve, which was included in triplicate on each plate using known concentration DNA from a control individual.

Relative telomere lengths were calculated using Ct values determined by QuantStudio software using standard curve efficiencies, and by Linregpcr, a software which uses individual sample reaction efficiencies to calculate Ct values. The Ct values of the telomere and RAG1 amplification determined by QuantStudio and Linregpcr were significantly correlated (Tel: cor = .61, *df* = 74, *p* = <.001, RAG1: cor = .94); however, the Ct values of the RAG1 amplification did differ significantly between the two methods (*t* = −4.42, *df* = 74, <.001). The relative telomere lengths calculated with the Ct values of the two different methods, QuantStudio and Linregpcr, were also significantly correlated (cor = .66, *df* = 74, *p* = <.001). The relative telomere lengths used in final models were calculated using the Ct values produced in Linregpcr, chosen due to the more precise method, and were used in all subsequent analyses. However, running the models with relative telomere lengths calculated using QuantStudio provided Ct values did not change the results or interpretation of the models (Table S3).

The ΔΔCt (or relative telomere length/RTL) was calculated as follows (Joglekar et al., 2020):

$$\begin{array}{c} \text{ΔΔCt}=\left(\text{Sample}\;\text{Average}\;\mathrm{RAG}1\;\mathrm{Ct}-\text{Sample}\;\text{Average}\;\text{Telomere}\;\mathrm{Ct}\right)\\ \;-\left(\text{Control}\;\text{Average}\;\mathrm{RAG}1\;\mathrm{Ct}-\text{Control}\;\text{Average}\;\text{Telomere}\;\mathrm{Ct}\right). \end{array}$$

### 2.3 Data analysis

#### 2.3.1 Does early‐life telomere length affect rate of senescence in reproductive traits?

All data analysis was performed in R version 3.5.2. Three models were constructed using glmmTMB (Brooks et al., 2017) to test the interaction effects of age and early‐life telomere length on clutch size (number of eggs), hatching success (proportion of eggs laid which hatched) and fledging success (proportion of chicks hatched which fledged; Table 1). All models included year, female identity and lay date (relative to the first lay date of the season) as random factors, the latter because of its previously found importance in the success of the hatchling to fledgling transition (de Villemereuil et al., 2019). All three models (Table 1) included early‐life telomere length, female age and lifespan as fixed effects. Early‐life telomere length and female age (in years) were tested as continuous variables and were both scaled using mean centring. Lifespan was included in all the models presented in Table 1 to control for selective disappearance (Nussey et al., 2006; van de Pol & Verhulst, 2006), that is, the fact that individual quality covaries with the likelihood that the individual will disappear from the population. Specifically, the selective disappearance hypothesis assumes a correlation between age of last reproduction and individual quality (Vaupel & Yashin, 1985). Diagnostics of models were checked using the DHARMa package (Hartig & Hartig, 2017), and the final three models (Table 1) were selected using AIC values (Tables S1 and S2). For data visualization and summary statistics, early‐life relative telomere lengths were categorized as ‘short’ or ‘long early‐life telomeres’ depending on whether they fell below or above the first quartile telomere length.

**TABLE 1:** Results of three separate generalized linear mixed models testing for the effect of early‐life telomere length (TL) on the relationship between female age and her output in three reproductive traits.

Factor	Clutch size			Hatching success			Fledging success		
	Estimate	SE	*p*	Estimate	SE	*p*	Estimate	SE	*p*
Age	0.025	0.03	**<.001**	−0.04	0.059	.5	0.12	0.12	.36
Age^2^	−0.011	0.002	**<.001**	−0.039	0.017	**<.05**	−0.003	0.05	.94
TL	−0.025	0.011	**.01**	−0.16	0.08	.05	−0.07	0.12	.54
Age^2^ × TL	0.0032	0.001	**<.01**	0.022	0.008	**<.01**	0.016	0.023	.48
Lifespan	−0.0024	0.0058	.68	0.064	0.045	.15	−0.054	0.06	.37
**Random effect**	**Variance**	**SD**		**Variance**	**SD**		**Variance**	**SD**	
Year	0.0015	0.038		1.33e‐10	1.2e‐5		0.26	0.51	
Female ID	0.0011	0.033		0.1	0.33		2.73e‐9	5.22e‐5	
Lay date	0.0015	0.039		5.34e‐9	7.3e‐5		0.036	0.19	

*Note*: Lifespan was included in the model as a fixed effect to control for selective disappearance. Year, female ID and lay date (relative to the first lay date of the season) were included as random effects to control for repeat measures from females across the lifespan, temporal variation across years and timing within the breeding season.

Abbreviations: SD, standard deviation; SE, standard error.

**FIGURE 1:**
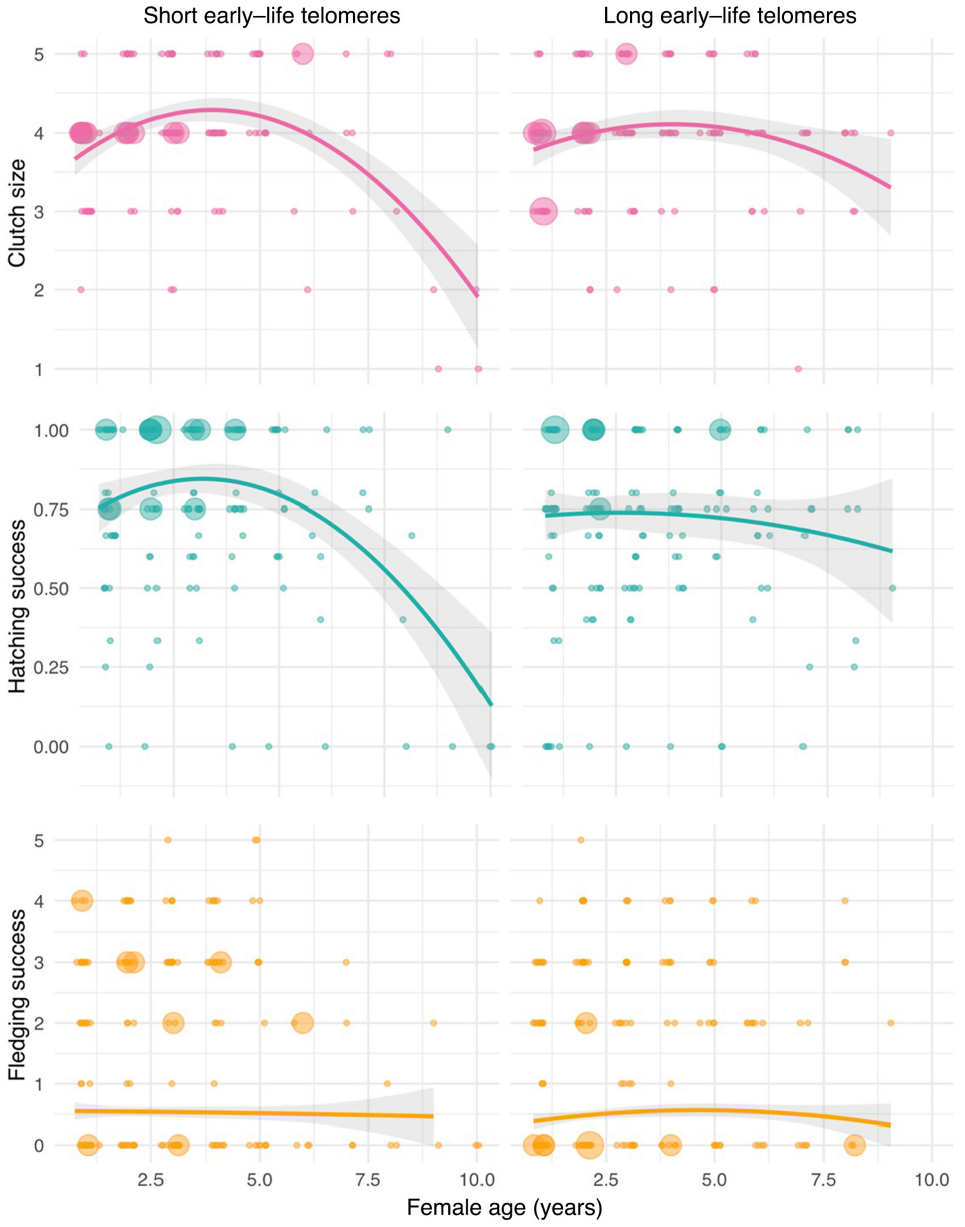
For data visualization purposes, early‐life telomere length was classed as ‘long’ or ‘short’ depending on whether it was above or below the 1st quartile. The 1st quartile was chosen after plotting female age against reproductive traits for all 4 quartiles of the range of early‐life telomere length, showing that the 1st quartile seemed to be the critical length (see Supporting Information for a plot of all 4 quartiles and details). Early‐life telomere length was analysed as a continuous variable in all statistical models. The area of the point/bubble increases when the sample size at those X,Y values is greater. The rate of decline in (a) clutch size and (b) hatching success with female age varies with early‐life relative telomere length. Females with very short telomeres (in the lowest quartile of the range) show faster rates of senescence in (a) clutch size and (b) hatching success. The rate of decline in fledging success with female age is not influenced by early‐life telomere length.

The effects of female age and early‐life relative telomere length on clutch size were tested using a com‐Poisson model for underdispersed count data. The effects of female age and early‐life relative telomere length on hatching success were tested using generalized linear mixed‐effects models with binomial or beta‐binomial errors. Linear, quadratic and breakpoint relationships between female age and hatching success were tested. Breakpoint models are regression models where the relationship between the independent and dependent variables is partitioned into two or more segments which are modelled separately by linear regression. The breakpoints were estimated at 2 and 5 years and identified visually by plotting the data (Figure 1). The effects of female age and early‐life relative telomere length on fledging success were tested with zero‐inflated binomial model due to the disproportionate number of clutches which fledged zero chicks. Male age was an additional random factor included in this model to control for the contribution of the male partner to provisioning. However, due to the correlation between female and male age in a pair, male age was removed from the final model.

Two linear models of clutch size and hatching success after the onset of senescence (postpeak performance) were also constructed using lme4. The age of peak clutch size/hatching success was the age (in years) with the highest mean clutch size/hatching success and was calculated separately for females with ‘short’ and ‘long’ early‐life telomeres. If two age categories had mean clutch size/hatching success which were not significantly different, determined by overlapped standard errors, we selected the first peak as the peak age for downstream postpeak analysis. Linear models of postpeak clutch size/hatching success were limited to data from females older than the age of peak clutch size/hatching success for the relative early‐life telomere lengths and included female ID, year and relative lay date as random factors.

#### 2.3.2 Does early‐life telomere length predict lifetime reproductive success?

The effect of early‐life relative telomere length on lifetime reproductive success was tested using glmmTMB (Brooks et al., 2017) to construct 4 separate generalized linear mixed models for lifetime number of: eggs laid, eggs hatched, chicks fledged and recruits. Recruits were classed as individuals who bred at least once. All 75 females included in this study were classified as ‘dead’ (not seen in the 2 years prior to the last breeding season included in the study); therefore, the measures of lifetime reproductive success are accurate. Models were constructed with female lifespan as a fixed effect to demonstrate the relative importance of a female's lifespan on lifetime reproductive success and the cohort in which a female fledged as a random effect, to control for the effect of breeding period on lifetime reproductive success. All models were of the Poisson or com‐Poisson family and the model for the lifetime number of fledglings and recruits produced included a zero‐inflation component due to the disproportionate number of females who produced no fledglings or recruits in their lifetime. Mean performance in clutch size, hatching success and fledgling success was calculated for all 75 females using all breeding events during the lifespan. The contribution of mean performance in reproductive traits and lifespan to lifetime fitness (total number of recruits) was estimated using Pearson's correlation coefficients.

#### 2.3.3 Does early‐life telomere length predict lifespan?

The effect of early‐life telomere length on the lifespan of females was tested using the glmmTMB package (Brooks et al., 2017) to construct a generalized linear mixed‐effects model of Gaussian family, and the model included the cohort of the female as a random effect to control for temporal differences in extrinsic conditions which may affect lifespan.

## 3 RESULTS

### 3.1 Clutch size

There was a significant interaction between age and early‐life relative telomere length on a female's clutch size (Table 1), with females with longer telomeres showing a slower decline in clutch size across their lifespan (Figure 1). To visualize this interaction, we categorized females as having either ‘short’ or ‘long’ early‐life relative telomeres depending on whether telomere length fell below or above the first quartile telomere length, respectively (see Figure S2 for a version of Figure 1 separated by quartile and justification; Figure 1). The best‐fitting model (Table S1) included age as a quadratic term and linear term, with a significant interaction between the quadratic effect of age and telomere length, indicating a significant effect of telomere length on the peak clutch size of females.

Females with ‘short’ early‐life relative telomere length reached a peak mean clutch size of 4.6 eggs at 3 (mean = 4.52, SE = 0.12) and 6 (mean = 4.57, SE = 0.22) years of age, followed by a steep decline (from 3 years of age onwards, slope = −0.26, from 6 years onwards, slope = −0.63). Females with ‘long’ early‐life relative telomere length reached a lower peak mean clutch size of 4.2 (SE = 0.14), at 3 years of age, followed by a shallower decline (slope = −0.036). Analysis of postpeak hatching success in females over 3 years of age revealed a significant negative effect of post‐peak female age on clutch size in females with short early‐life telomeres (*df* = 1, Chisq = 11.71, *p* = .00062), but a lack of significant effect in females with long early‐life telomeres (*df* = 1, Chisq = 0.17, *p* = .68).

### 3.2 Hatching success

Females with longer telomere lengths show a slower senescence in hatching success during their lifespan (Figure 1). The best‐fitting model (Table S2) showed a significant interaction between the quadratic age term and telomere length on hatching success (Table 1), indicating a significant effect of early‐life telomere length on the peak hatching success of females.

Females with short early‐life telomeres peaked in their mean hatching success at 4 years of age, with 86% (SE = 0.05) hatching success, followed by a steep decline (slope = −0.1), females with long early‐life telomeres peaked in their mean hatching success at 2 years of age with 80% (SE = 0.04) hatching success, followed by a much shallower decline (slope = −0.017). Analysis of postpeak hatching success including females which were older than 4 years of age if they possessed short early‐life telomeres, and females over 2 years of age if they possessed long early‐life telomeres revealed a significant negative effect of postpeak female age on hatching success in females with short early‐life telomeres (*df* = 1, Chisq = 9.1, *p* = .0026). There was a lack of significant effect of postpeak female age on hatching success in females with long early‐life telomeres (*df* = 1, Chisq = 1.49, *p* = .22).

### 3.3 Fledging success

There was no significant effect of a female's early‐life relative telomere length on the fledging success rate of her chicks, nor was there any strong evidence of an effect of female age or lifespan. There also was no significant interaction between early‐life relative telomere length and age on fledging success, suggesting that early‐life telomere length does not influence the rate of senescence of this trait (Table 1). We attempted to control for the age of the social male partner in this model to account for the fact that a decline in fledgling success due to maternal senescence may be offset by the provisioning efforts of the male social partner. However, there was a significant correlation between the ages of the female and male in a social pair (*df* = 1294, *r* = .17, *p* = <.001), leading to collinearity and a lack of convergence in the model. The correlation between ages of females and males in social pairs suggests that there is assortative pairing by age in this population of hihi, as there is an absence of mate fidelity. The potential effect of male age on fledging success may have therefore confounded our ability to detect an effect of female senescence in this reproductive trait.

### 3.4 Lifetime reproductive success

We found no significant effect of early‐life telomere length on any measures of lifetime reproductive success (Table 2). Lifetime reproductive success was instead driven by a female's lifespan (Table 3). The lack of a significant effect of early‐life telomere length on lifetime reproductive success, despite the significant impact on senescence of two key reproductive traits suggests that females with relatively ‘long’ and ‘short’ early‐life telomere lengths may be adopting alternative reproductive strategies. Those with shorter telomeres may invest more strongly prior to senescence, producing a greater number of recruits in their first few breeding years before senescent decline, whereas those with relatively long telomeres may produce fewer recruits per year but over a longer period. This theory is suggested by visualization of the data (Figure S3) and could be supported by an analysis of recruit production, but due to a large proportion of females producing no recruits every year, this requires a larger sample size than was available in this study.

**TABLE 2:** Results presented below are from four separate models with four measures of lifetime reproductive output of females as response variables, with both early‐life telomere length (TL) and lifespan included as fixed effects and fledging cohort of the female as a random effect.

~TL + lifespan	Early‐life telomere length			Lifespan		
	Estimate	SE	*p*	Estimate	SE	*p*
Lifetime eggs laid	−0.01	0.045	.81	0.27	0.021	**<.001**
Lifetime eggs hatched	−0.032	0.045	.48	0.27	0.022	**<.001**
Lifetime chicks fledged	−0.014	0.042	.73	0.21	0.02	**<.001**
Lifetime recruits	−0.14	0.14	.31	0.24	0.078	**.0025**

*Note*: *p* values <0.05 were in bold to highlight their statistical significance.

**TABLE 3:** Contribution of mean performance and total lifetime productiveness in three reproductive traits to lifetime fitness measured with Pearson's correlations.

Correlation with lifetime fitness (recruits)	*r*	*p*
Clutch size	.03	.81
Hatching success	.055	.63
Fledging success	.15	**.2**
Lifespan	.39	**<.001**

*Note*: Lifetime fitness is estimated as the number of recruits (breeding individuals) produced in a lifetime.

*p* values <0.05 were in bold to highlight their statistical significance.

### 3.5 Lifespan

Early‐life telomere length did not have a significant effect on the lifespan of females (estimate = −0.17, SE = 0.21, *p* = .44).

## 4 DISCUSSION

Here, using long‐term data on females of an intensively monitored population of a threatened passerine bird (*Notiomystis cincta*) we demonstrate that early‐life relative telomere length predicts senescence in key reproductive traits. Females with shorter early‐life telomeres experienced a significant, steeper postpeak decline in both clutch size and hatching success, compared with females with longer early‐life telomeres. However, early‐life telomere length does not predict the rate of senescence in fledging success rate, nor an individual's lifespan. In turn, early‐life telomere length does not predict lifetime reproductive success, which is significantly influenced by an individual's lifespan and the fledging success rate of their nestlings rather than their clutch size or hatching success rate. These results suggest that females may adopt alternative reproductive strategies based on their early‐life conditions, of which their telomere length may be representative.

### 4.1 Early‐life telomere length predicts senescence rate in multiple female reproductive traits

We found that a female's early‐life telomere length predicts the presence of significant senescence in both clutch size and hatching success, with shorter telomeres being associated with more rapid senescence in these two reproductive traits. This confirms our original prediction, based on the ‘trade‐off’ concept of telomere attrition and the disposable soma theory of ageing, that short telomeres in early life are equal to less ‘trading power’, resulting in a faster rate of senescence. The disposable soma theory states that reproductive investment leads to a trade‐off with bodily maintenance (Kirkwood & Holliday, 1979); telomere attrition provides a possible mechanism for this trade‐off. In this system, early‐life telomere length may be influencing reproductive strategy; females with short early‐life telomere length have a higher peak in reproductive success in clutch size and hatching in early life followed by a faster rate of decline. This result aligns with cross‐species and intraspecific cross‐population studies of the fast‐slow life‐history continuum, which show that faster life history predicts faster and earlier onset of senescence (Cayuela et al., 2020; Giaimo & Traulsen, 2019; Jones et al., 2008). Within‐population studies of intraspecific variation in the fast‐slow life‐history continuum are rare, and although the results of this study suggest that early‐life telomere length may be a good indicator of reproductive trade‐offs across the lifespan, the same is not necessarily true for lifespan and lifetime fitness. It appears that females with short early‐life telomeres may also have higher annual fitness in early life than females with long early‐life telomeres (Figure S3); however, this effect was not testable due to a high proportion of zero values for annual fitness, requiring a larger sample size. Our finding that early‐life telomere length can predict rates of reproductive senescence is supported by previous findings, which show that early‐life telomere length can predict lifetime reproductive success through its effect on lifespan (Eastwood et al., 2019) and that increased reproduction in early life can lead to faster senescence in later life (Nussey et al., 2006; Reed et al., 2008). However, ours is the first study to demonstrate a link between early‐life telomere length and rates of senescence in specific reproductive traits, independent of lifespan.

The proposed mechanisms linking telomeres to reproductive ageing have primarily been researched in humans and model organisms such as mice (Chico‐Sordo et al., 2021; Kalmbach et al., 2013). Short telomeres can disrupt meiosis (Polonio et al., 2020) and increase the likelihood of embryo fragmentation in humans (Keefe et al., 2005), which in birds may feasibly lead to reduced clutch size or higher rates of hatching failure. However, it is worth considering that here we measure early‐life telomere length from blood samples and not reproductive tissue. In humans, tissue type can significantly impact the telomere length measured (Demanelis et al., 2020) although, telomere length measurements across five tissue types in painted dragon lizards were significantly correlated (Rollings et al., 2019). Little is known about how telomere length and attrition vary across cell and tissue types in birds, although it would be reasonable to hypothesise that the telomere dynamics in cells of reproductive tissues may be more directly subject to DNA damage associated with reproduction. Lifetime reproductive success is not predicted by early‐life telomere length.

Despite some indication that female hihi adopt differing reproductive strategies depending on early‐life telomere length, this does not appear to influence lifetime reproductive success. This could be because lifetime reproductive success is primarily determined by fledgling success, for which we found no evidence of an effect of early‐life telomere length. It has previously been shown, using simulated data, that the fitness cost of reproductive failure at early stages is significantly lower than at late stages (Lemaître & Gaillard, 2017). Alternatively, the small effect size of the interaction between early‐life telomere length and age on reproductive traits may suggest that the effect of senescence is not large enough to significantly impact lifetime fitness, as has previously been found in a study on great tits (*Parus major*; Bouwhuis et al., 2012). Nevertheless, our results question the recent discussion of telomere length as biomarker of fitness (Eastwood et al., 2022), demonstrating that this idea is not universally applicable.

A female hihi's social male partner provisions chicks in the nest at variable rates depending on male age and other factors (Ewen & Armstrong, 2000; Low et al., 2006). Fledging success is therefore likely also influenced by male‐mediated factors, including paternal age. Sexes can differ in patterns of actuarial senescence (Loison et al., 1999) and reproduction‐induced telomere shortening rates (Sudyka et al., 2014). Male hihi show variable reproductive success with age (Brekke et al., 2015), creating the potential for females to offset the effects of their own reproductive senescence by pairing with younger males. However, we found a significant correlation between the age of females and males in social pairs, indicating assortative mating by age, which potentially confounds our analysis of female senescence in fledging success.

Several environmental factors not tested here are more likely to influence fledgling success than clutch size or hatching success, potentially masking any effects of female early‐life telomere length on this trait. Rainfall (Capilla‐Lasheras et al., 2021), temperature (Chase et al., 2005) and storm events (Wallace et al., 2016) can impact the number of successful fledglings from a nest due to their effects on insect availability and activity (Cox et al., 2019); an important food source for hihi nestlings. It has been shown that extreme temperatures cause nestling starvation and mortality in hihi, (Rippon et al., 2011), but not hatching failure (Low & Pärt, 2009). More research is needed to account for factors such as environmental variations and male age/quality when studying female senescence in fledging success.

Female lifespan was by far the largest contributor to lifetime reproductive success. Surprisingly, female lifespan was not predicted by early‐life telomere length in our study, which may also explain the lack of relationship between early‐life telomere length and lifetime reproductive success. Our results contradict the very few studies which have found a relationship between early‐life telomere length and lifetime reproductive success (Eastwood et al., 2019; Heidinger et al., 2021) and suggest that early‐life telomere length is not under selection in females of this species. Instead, we hypothesise that the main drivers of early‐life telomere length in this system are perhaps developmental conditions and maternal effects, as has been found in other species (Boonekamp, Mulder, et al., 2014; Cram et al., 2017; Stier et al., 2020; van Lieshout et al., 2019).

The ‘silver spoon’ hypothesis predicts that individuals who experience favourable early‐life conditions, which may be reflected in their early‐life telomere length (Cram et al., 2017), have increased lifetime reproductive success (Grafen, 1988). Previous studies have provided support for the silver spoon hypothesis, with favourable developmental conditions being shown to increase lifetime reproductive success (Pigeon et al., 2017; Van De Pol et al., 2006) and reproductive investment in early life, albeit at the expense of accelerated senescence (Spagopoulou et al., 2020). However, we found no association between early‐life telomere length and lifetime reproductive success, suggesting that longer early‐life telomeres are instead associated with a reduced rate of reproductive senescence. This aligns with evidence that favourable conditions in early life lead to slower rates of reproductive senescence (Cooper & Kruuk, 2018; Nussey et al., 2007). However, further research is needed to determine the influence of developmental conditions on early‐life telomere length in hihi before conclusions are made about the impact of the ‘silver spoon’ on reproductive senescence in this species.

## 5 CONCLUSIONS

There are few studies where the link between telomeres and reproduction is considered across an individual's lifespan (reviewed in Sudyka, 2019). In this study, we have demonstrated, using data on lifetime output in key reproductive traits, that females experience different rates of reproductive senescence depending on their early‐life telomere length. The lack of an effect of early‐life telomere length on lifespan and lifetime reproductive success, despite the clear effect on rate of senescence in reproductive traits, raises the possibility of reproductive strategies that differ with early‐life telomere length and questions the ubiquity of telomere length as an indicator of future lifespan and lifetime fitness. Although it has been previously suggested that telomeres shorten as a result of high reproductive investment (Bauch et al., 2013; Bichet et al., 2020; Ouyang et al., 2016), our results suggest that variation in reproductive traits is partially predetermined by an individual's early‐life telomere length.

## AUTHOR CONTRIBUTIONS

FM carried out the experimental work and analysis, JGE oversaw field data and sample collection, and PB oversaw genotyping and pedigree development. FM wrote the manuscript with support from PB, NH and MJPS. NH conceived the study with support from PB. All authors reviewed and approved the final manuscript.

## CONFLICT OF INTEREST STATEMENT

The authors declare no conflicts of interest.

## BENEFIT‐SHARING STATEMENT

The sample collection was carried out in Tiritiri Matangi, New Zealand. In order to carry out data collection, access and data rights were agreed with Ngati Manuhiri Settlement Trust as Mana Whenua and Kaitiaki of Tiritiri Matangi and its taonga, including hihi. This and associated research will be disseminated to Ngati Manuhiri in the form of monthly research updates and an annual report from the Hihi Recovery Group.

A research collaboration exists with the Hihi Recovery Group, which is funded by the Hihi Conservation Charitable Trust, a New Zealand charitable trust with the purpose of conserving the species. Dr John Ewen is chair of the Hihi Recovery Group and Trustee of the Hihi Conservation Charitable Trust and is included as a co‐author. The funding associated with this work has contributed towards funding the salary of a conservation officer working for the Hihi Recovery Group and solely funded a local research assistant for two field seasons.

## Supporting information


Appendix S1.

## Data Availability

Scripts used to generate the analyses presented in the paper will be made publicly available at https://github.com/fmorland/hihitelomeres.git upon acceptance of the manuscript (Morland et al., 2023). Hihi are of cultural significance to the indigenous people of Aotearoa New Zealand, the Māori, and are considered a taonga (treasured) species whose whakapapa (genealogy) is intricately tied to that of Māori. For this reason, the telomere data and associated pedigree for hihi will be made available by request on the recommendation of Ngāti Manuhiri, the iwi (tribe) that affiliates as kaitiaki (guardians) for hihi. To obtain contact details for the iwi, please contact Dr Patricia Brekke at patricia.brekke@ioz.ac.uk. This process is necessary in order to maintain current permit stipulations and in agreement with NZ's laws regarding the Nagoya and Waitangi treaty.
