# Supplementary material for: Early‐life telomere length predicts life‐history strategy and reproductive senescence in a threatened wild songbird
Source: Mol Ecol. 2023 May 12;32(14):4031–43. doi: 10.1111/mec.16981 (PMC10947174; doi:10.1111/mec.16981)
Supplement: Supplementary file 1 — Appendix S1. [file MEC-32-4031-s001.docx]

**Supplementary Information** - Early-life telomere length predicts life-history strategy and reproductive senescence in a threatened wild songbird

*Supplementary Table 1. AIC values were used to select the best model for the effect of female age and female ID on clutch size, the best fitting model included a quadratic relationship between age and clutch size. Linear, quadratic and breakpoint models were tested but breakpoint models failed to converge. All models had a Conway-Maxwell Poisson or Poisson error distribution.*

| Model formula for clutch size | AIC | ΔAIC |
| --- | --- | --- |
| clutch size ~ age^2^ * TL + age + lifespan + (1 \| year) + (1 \| ID) + (1 \| lay date) | 787.9 | 0 |
| clutch size ~ age^2^ + TL * age + lifespan + (1 \| year) + (1 \| ID) + (1 \| lay date) | 789.2 | 1.3 |
| clutch size ~ age^2^ * TL * age + lifespan + (1 \| year) + (1 \| ID) + (1 \| lay date) | 789.2 | 1.3 |
| clutch size ~ age^2^ + TL + age + lifespan + (1 \| year) + (1 \| ID) + (1 \| lay date) | 794.6 | 6.7 |

*Supplementary Table 2. AIC values were used to select the best model for the effect of female age and female early-life telomere length on hatching success. Linear, quadratic and breakpoint models were tested. All models had either betabinomial or binomial error distributions.*

| Model formula | AIC | ΔAIC |
| --- | --- | --- |
| hatching success ~ age^2^ * RTL + age + lifespan + (1 \| year) + (1 \| ID) + (1 \| lay date) | 883.3 | 0 |
| hatching success ~ age^2^ + RTL * age + lifespan + (1 \| year) + (1 \| ID) + (1 \| lay date) | 887.0 | 3.7 |
| hatching success ~ age^2^ + RTL + age + lifespan + (1 \| year) + (1 \| ID) + (1 \| lay date) | 889.8 | 6.5 |
| hatching success ~ age (with breakpoints at 2 and 5 years)*ΔRTL + lifespan + (1 \| year) + (1 \| ID) + (1 \| lay date) | 902.8 | 19.5 |
| hatching success ~ age (with breakpoint at 0.42 years)*ΔRTL+ lifespan + (1 \| year) + (1 \| ID) + (1 \| lay date) | 911.9 | 28.6 |

Supplementary Table 3. The results of three separate generalised linear mixed models testing for the effect of early-life telomere length (TL), as measured using Quantstudio provided Ct values, on the relationship between female age and her output in three reproductive traits. Lifespan was included in the model as a fixed effect to control for selective disappearance. Year, female ID and lay date (relative to the first lay date of the season) were included as random effects to control for repeat measures from females across the lifespan, temporal variation across years and timing within the breeding season.

|  | Clutch Size | | | Hatching Success | | | Fledging Success | | | |
| --- | --- | --- | --- | --- | --- | --- | --- | --- | --- | --- |
| Factor | Estimate | S.E | P | Estimate | S.E | P | Estimate | S.E | P |  |
| Age | 0.026 | 0.0088 | **<0.01** | -0.041 | 0.06 | 0.51 | 0.12 | 0.12 | 0.35 |  |
| Age^2^ | -0.013 | 0.0024 | **<0.001** | -0.038 | 0.046 | **0.034** | -0.0048 | 0.053 | 0.93 |  |
| TL | -0.016 | 0.0058 | **<0.01** | -0.044 | 0.045 | 0.33 | -1.08 | 0.103 | 0.3 |  |
| Age^2^*TL | 0.0025 | 0.0009 | **<0.01** | 0.015 | 0.007 | **0.029** | 0.015 | 0.02 | 0.44 |  |
| Lifespan | -0.002 | 0.0061 | 0.75 | 0.069 | 0.049 | 0.16 | 0.064 | 0.062 | 0.3 |  |


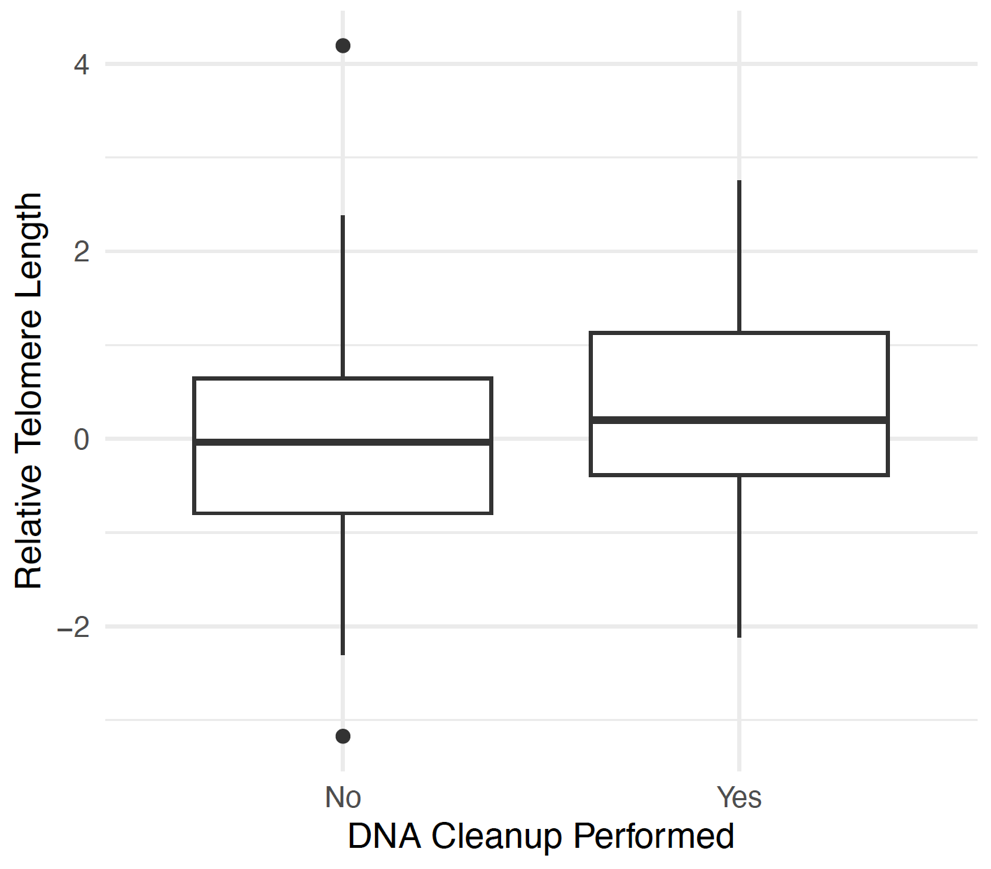


Supplementary Figure 1. The relative telomere length of samples which underwent a magnetic bead cleaning process (n = 26) did not significantly differ from those which did not undergo a bead cleaning process (n=49), t=1.18, df = 54.23, p = 0.26).


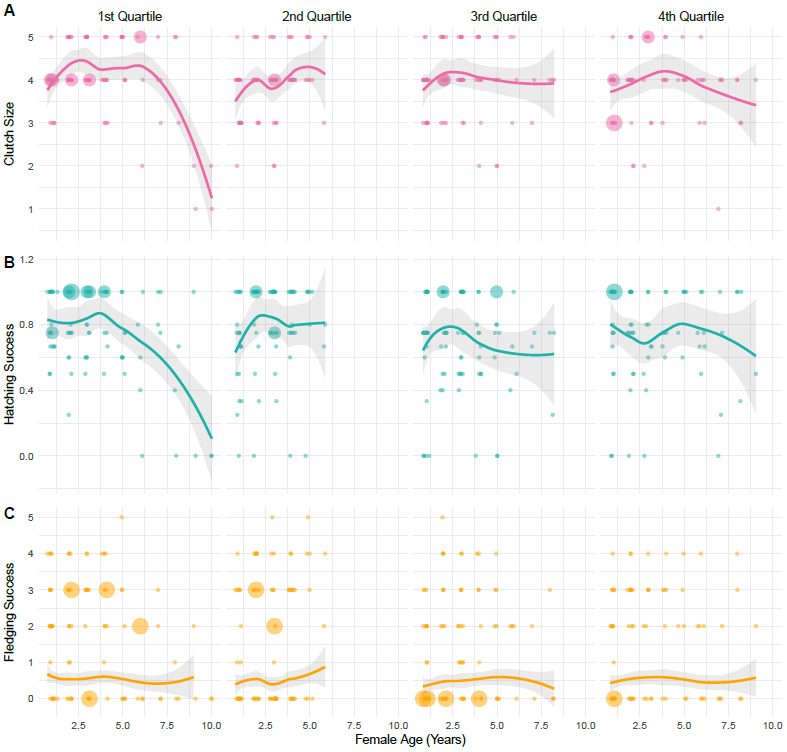


Supplementary Figure 2. The rate of decline in A) clutch size and B) hatching success with female age varies with early-life relative telomere length. The plot is divided here into quartiles of early-life telomere length: 1^st^ quartile = min (-3.17) to 1^st^ quartile (-0.75), 2^nd^ quartile = 1^st^ quartile to the median (0.127), 3^rd^ quartile = median to 3^rd^ quartile (0.81) and 4^th^quartile = 3^rd^ quartile to the max early life telomere length (3.1). Females with very short telomeres (in the lowest quartile of the range) show faster rates of senescence in A) clutch size and B) hatching success. For the first quartile appears to be the “tipping point” for the effects we see of early-telomere length on reproductive senescence, therefore the 1^st^ quartile was selected at the separation point in the main text figure.


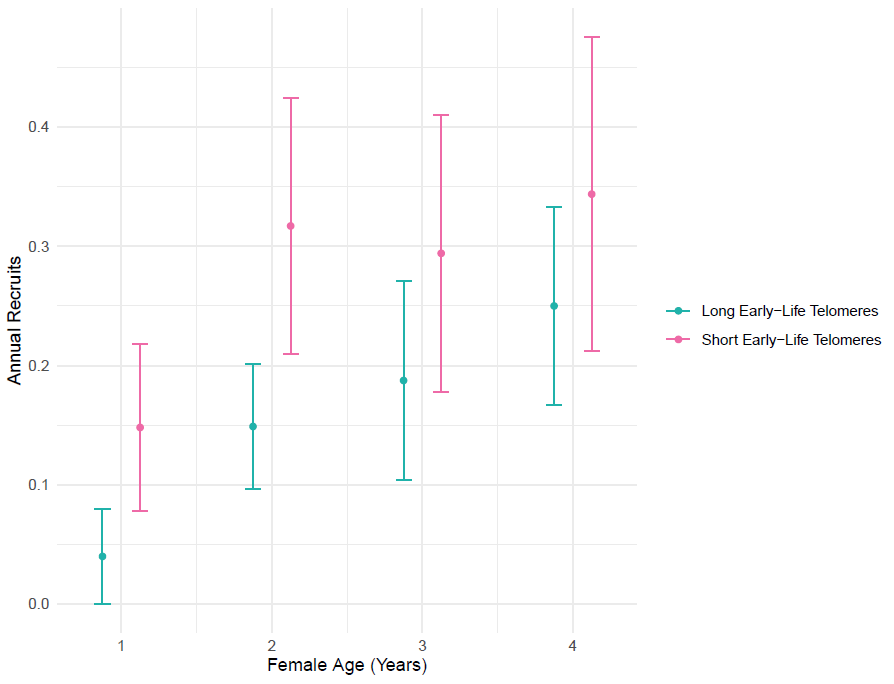


Supplementary Figure 3. The mean annual recruits produced by females with "short" and "long" early-life telomere lengths (classified as below or above the first quartile respectively, due to this length being a significant "tipping point" for an effect on senesence to be observed – see supplementary Figure 2). Females with short early-life telomere length experience a faster senesence in clutch size and hatching success and appear here to have higher reproductive output in early life, particularly the first two breeding seasons. However, this effect was not testable due to the sample size.
